# A novel null mutation in the pyruvate dehydrogenase phosphatase catalytic subunit gene (*PDP1*) causing pyruvate dehydrogenase complex deficiency

**DOI:** 10.1002/jmd2.12054

**Published:** 2019-06-17

**Authors:** Jirair K. Bedoyan, Leah Hecht, Shulin Zhang, Stacey Tarrant, Ann Bergin, Didem Demirbas, Edward Yang, Ha Kyung Shin, George J. Grahame, Suzanne D. DeBrosse, Charles L. Hoppel, Douglas S. Kerr, Gerard T. Berry

**Affiliations:** ^1^ Department of Genetics and Genome Sciences Case Western Reserve University (CWRU) Cleveland Ohio; ^2^ Pediatrics Case Western Reserve University (CWRU) Cleveland Ohio; ^3^ Center for Human Genetics University Hospitals Cleveland Medical Center (UHCMC) Cleveland Ohio; ^4^ Center for Inherited Disorders of Energy Metabolism (CIDEM) University Hospitals Cleveland Medical Center (UHCMC) Cleveland Ohio; ^5^ Division of Genetics and Genomics, The Manton Center for Orphan Disease Research Boston Children's Hospital, Harvard Medical School Boston Massachusetts; ^6^ Pathology and Laboratory Medicine University of Kentucky Lexington Kentucky; ^7^ Department of Neurology Boston Children's Hospital, Harvard Medical School Boston Massachusetts; ^8^ Radiology Boston Children's Hospital, Harvard Medical School Boston Massachusetts; ^9^ School of Medicine Case Western Reserve University (CWRU) Cleveland Ohio; ^10^ Medicine Case Western Reserve University (CWRU) Cleveland Ohio; ^11^ Pharmacology Case Western Reserve University (CWRU) Cleveland Ohio

**Keywords:** branched‐chain 2‐ketoacid dehydrogenase, developmental delay, lactic acidosis, PDP1, pyruvate dehydrogenase complex deficiency, pyruvate dehydrogenase phosphatase deficiency

## Abstract

Congenital lactic acidosis due to pyruvate dehydrogenase phosphatase (PDP) deficiency is very rare. PDP regulates pyruvate dehydrogenase complex (PDC) and defective PDP leads to PDC deficiency. We report a case with functional PDC deficiency with low activated (+dichloroacetate) and inactivated (+fluoride) PDC activities in lymphocytes and fibroblasts, normal activity of other mitochondrial enzymes in fibroblasts, and novel biallelic frameshift mutation in the *PDP1* gene, c.575dupT (p.L192FfsX5), with absent PDP1 product in fibroblasts. Unexpectedly, the patient also had low branched‐chain 2‐ketoacid dehydrogenase (BCKDH) activity in fibroblasts with slight elevation of branched‐chain amino acids in plasma and ketoacids in urine but with no pathogenic mutations in the enzymes of BCKDH, which could suggest shared regulatory function of PDC and BCKDH in fibroblasts, potentially in other tissues or cell types as well, but this remains to be determined. The clinical presentation of this patient overlaps that of other patients with primary‐specific PDC deficiency, with neonatal/infantile and childhood lactic acidosis, normal lactate to pyruvate ratio, elevated plasma alanine, delayed psychomotor development, epileptic encephalopathy, feeding difficulties, and hypotonia. This patient exhibited marked improvement of overall development following initiation of ketogenic diet at 31 months of age. To the best of our knowledge, this is the fourth case of functional PDC deficiency with a defined mutation in *PDP1*.

**Synopsis:**

Pyruvate dehydrogenase phosphatase (PDP) regulates pyruvate dehydrogenase complex (PDC) and defective PDP due to *PDP1* mutations leads to PDC deficiency and congenital lactic acidosis.

**Abbreviations**
BCKDH: branched‐chain 2‐ketoacid dehydrogenase
DCA: dichloroacetate
KD: ketogenic diet
KDC: 2‐ketoglutarate dehydrogenase complex
PDC: pyruvate dehydrogenase complex
PDP: pyruvate dehydrogenase phosphatase

## 1 INTRODUCTION

The mitochondrial multienzyme pyruvate dehydrogenase complex (PDC) irreversibly catalyzes oxidative decarboxylation of pyruvate into acetyl‐CoA as the primary substrate from carbohydrate for the tricarboxylic acid (TCA) cycle and oxidative phosphorylation. Defective complex function results in increased cellular pyruvate levels which leads to elevated lactate and alanine levels. PDC is comprised of four core catalytic subunits (E1α, E1β, E2, and E3 encoded by the *PDHA1*, *PDHB*, *DLAT*, and *DLD* genes, respectively) and a structural protein (E3BP/*PDHX*). E3 is also a part of other 2‐keto acid dehydrogenases (eg, BCKDH and 2‐ketoglutarate dehydrogenase) as are the three cofactors (thiamine pyrophosphate, TPP; covalently bound lipoate; and flavin adenine dinucleotide, FAD).

PDC activity is highly regulated; it is inactivated by phosphorylation of serine sites on E1α by one of four pyruvate dehydrogenase kinases (PDKs: PDK1, PDK2, PDK3, and PDK4), while activation of PDC is achieved by removal of these phosphates from E1α by two pyruvate dehydrogenase phosphatases (PDPs: PDP1 and PDP2).^1^ The lipoate cofactor plays a critical role in stabilizing and regulating PDC function.^2^ PDC also is glutathionylated on E2, and this glutathionylation decreases reactive oxygen species (ROS) production when pyruvate is being oxidized, while depletion of glutathione leads to increased ROS production from PDC.^3^ Glutathione reductase (GRX2) regulates the reversible glutathionylation, which also is important for PDC activity.^3^ Sirtuin 4 (SIRT4) regulates PDC function through its lipoamidase activity that cleaves the lipoyl moiety from E2.^4^ Defective biosynthesis or mitochondrial transport of cofactors (eg, thiamine) or substrates (eg, pyruvate) can result in functional PDC deficiency.^5^ PDC also can translocate from the mitochondria to the nucleus during cell‐cycle progression, generating a nuclear pool of acetyl‐CoA from pyruvate and increasing the acetylation of core histones important for S phase entry and expression of damage response genes among others.^6^ In summary, PDC is a regulated mitochondrial multienzyme complex crucial for oxidation of carbohydrate for energy production, but with other “moonlighting” role(s) in cell‐cycle progression and potentially cellular differentiation and proliferation.

Two isoforms of PDP are known.^7^ One isoform is a heterodimer consisting of a catalytically activity subunit (PDP1c, encoded by *PDP1*) and a larger regulatory FAD‐associated subunit (PDPr, encoded by *PDP3*, also known as *PDPR*). PDPr regulates the activity of PDP1c by blocking and distorting its active site. The other isoform consists of only the phosphatase catalytic subunit encoded by PDP2.^8^ A regulatory subunit is not required for PDP2, which is 50× less active than PDP1.^9^ PDP1 and PDP2 are ubiquitously expressed in most tissues but both are highly expressed in brain although PDP1 protein is about sevenfold higher than PDP2; PDP1 expression predominates in skeletal and cardiac muscles, adrenal glands and testis, while PDP2 in liver, adipocytes, thyroid, ovary, and kidney (HPA RNA‐seq normal tissues profiles from NCBI Gene ID: 54704 and 57 546 for *PDP1* and *PDP2*, respectively).^8^, ^10^ PDP1 requires both calcium and interaction with the lipoyl domain of the E2 subunit of PDC for optimal activity, while PDP2 requires neither.^7^, ^11^ Although both isoforms are magnesium dependent, PDP2 requires 10‐fold higher concentration for activity than PDP1.^8^ Insulin and insulin mimetic agents activate PDP in a dose‐dependent manner.^12^ Thus, PDC activity is regulated by PDP, which itself is regulated by calcium, magnesium, and insulin concentrations in cells and tissues.

Several cases with “atypical” PDC deficiency attributed to a defect in phosphatase activation of the PDC complex have been reported, without identifying pathogenic variants in genes associated with phosphatase deficiency (*PDP1*, *PDP2*, and *PDPR*) explaining the functional PDC deficiency.^13^, ^14^, ^15^, ^16^, ^17^, ^18^, ^19^ Maj et al^7^ review these reported cases and question whether the cases truly reflect patients with PDP deficiency or demonstrate inefficient activation of PDC for reasons other than a defective PDP.

Consequently, the reported cases of PDC deficiency due to confirmed mutations in either *PDP1*, *PDP2*, or *PDPR* are very limited. The clinical presentation of PDP deficiencies (due to mutations in *PDP1*, *PDP2*, or *PDPR*) not surprisingly overlaps that of PDC deficiencies, with neonatal/infantile and childhood lactic acidosis, encephalopathy, brain abnormalities, seizures, feeding difficulties, hypotonia, cardiomyopathy, corneal clouding, and delayed psychomotor development reported to date, although cardiomyopathy and corneal clouding are not typical of primary PDC deficiency.^9^, ^13^, ^15^, ^18^, ^20^, ^21^ We report a case of PDC deficiency due to a novel null mutation in *PDP1* presenting with lactic acidosis, delayed psychomotor development, and epilepsy with review of literature of cases with confirmed *PDP1* mutations.

## 2 CASE REPORT

This male proband was born full term, 3.4‐kg birth weight to a G_1_P_0‐1_ mother by C‐section due to prolonged labor, failure to progress, and fetal distress. Pregnancy was complicated by vaginal bleeding requiring bed rest. Perinatal course was notable for a 5‐day NICU stay for oxygen support. He was noted to be hypotonic from birth. Subsequently, there was failure to thrive. He did not sit until a year of age, was not walking at 20 months of age, and early on had limited physical (motor) endurance. Seizures began at 8 months of age with noted pallor, rigidity, bruxism, drooling and perioral cyanosis, and recurred with addition of eyelid closure, and enhanced hypotonia. He would be unresponsive during the episodes, which usually lasted between 5 and 10 minutes. His seizures ceased after initiating Keppra, with last seizure being at 14 months of age. At 15 months of age, brain MRI suggested a subtle region of increased FLAIR/T2 signal in the right frontal subcortical white matter, a small transmantle cortical dysplasia could not be ruled out. There were prominent CSF spaces around the cerebellum suggestive of cerebellar volume loss. He later developed reactive airways disease requiring Pulmicort and then Flovent. He also was noted to have an everted and pronated left foot and everted right foot.

At 25 months of age, his weight, length, and head circumference were 10.8 kg (5th percentile), 81.9 cm (5th percentile), and 48 cm (28th percentile), respectively. Metabolic testing showed lactic acidosis, elevated pyruvate with normal lactate to pyruvate ratio of 12, and abnormal findings on urine organic acids that included large amounts of 2‐keto‐isocaproic and 2‐keto‐isovaleric acids and small amounts of 2‐keto‐3‐methylvaleric and 2‐hydroxyisovaleric acids. Levels of methylmalonic acid in serum and creatine in plasma were normal. A chromosomal microarray analysis revealed a loss of heterozygosity (LOH) of about 16.3 Mb involving 10p11.2‐q11.23 (Chr10:33,865,952‐50,141,332, maximum breakpoint coordinates;hg19). At 28 months of age, a repeat brain MRI (Figure S1) showed abnormal T2 hyperintensity and diffusion restriction within the globus pallidus with subtle signal abnormality within the medial caudate heads and the posterior left putamen. Patchy T2 hyperintensity was also noted in the left parietal deep white matter, consistent with nonspecific gliosis. Signal and morphology of the brain were otherwise normal. Specifically, the myelination pattern was normal for age, and a congenital malformation (eg, callosal dysgenesis) was not identified. The ADC was dark in the globus pallidus bilaterally. Short and intermediate echo time MR spectroscopy revealed no detectable lactate, pyruvate, or branched‐chain ketoacid peaks.

He was started on an oral 3:1 (grams of fats to grams of carbohydrates plus proteins) ketogenic diet (KD) at 31 months of age once PDC deficiency was diagnosed and ketosis was adequately maintained (plasma beta‐hydroxybutyrate between 4.5 and 5.5 mM). His physical endurance markedly improved while on KD. However, due to continued feeding difficulty and food refusal, he would exhibit neurological dysfunction such as tremor, increased ataxia, and decreased alertness when unable to maintain adequate ketosis. About 15 months after KD initiation, a gastrostomy tube was placed to provide 75% to 100% of energy needs by ketogenic formula, resulting in the ability to maintain a more consistent level of ketosis. He required physical, occupational and speech therapies with placement in special education classes at school. His growth and development significantly improved while on KD, and KD stringency was reduced to 2.5:1 with advanced age. Although he exhibited speech delay early in development, by 7 years of age he spoke fluent English and Spanish, and attended class for children with special needs. Although he missed motor milestones and exhibited motor delay, at 7 years of age he was able to jump and attempted to climb stairs, but continued to need scheduled occupational, physical, and speech therapies. Despite developmental improvement, he continues to have poor oral intake and food refusal. When on KD, plasma alanine and proline were within their respective reference ranges, but the branched‐chain amino acids remained slightly above their respective reference ranges, but allo‐isoleucine was not noted on plasma amino acids. The highest levels of plasma branched‐chain amino acids noted at 3.5 years of age were valine 569 μM (RR 105‐407), isoleucine 206 μM (RR 24‐124) and leucine 331 μM (RR 56‐228). He also was maintained on thiamine, L‐carnitine, calcium and vitamin D3, polycitra‐K or baking soda, multivitamin, and Keppra. Family history was non‐contributory with no reported consanguinity. Parents were of Hispanic origin.

## 3 MATERIALS AND METHODS

### 3.1 Next‐generation sequencing analysis

Coding exons and immediate flanking intronic regions of 23 genes associated with pyruvate metabolism^22^ were enriched by PCR and subsequently sequenced by next‐generation sequencing (NGS) using an Ion Torrent PGM system. Sequencing FastQ files were aligned to human genome reference sequence version GRCh 38/hg38. Variant calling were performed using Torrent Variant Caller (V4.4.2.1). Based on our validation data, approximately 98% of variants detected by Sanger sequence can be reliably identified by this platform. Regions <20× coverage were completed by Sanger sequencing. Clinically significant pathogenic or likely pathogenic variants were confirmed by Sanger sequencing using standard methods. Sequencing and deletion/duplication analyses of the *BCKDHA*, *BCKDHB*, *DBT*, and *PPM1K* genes using NGS technology were performed at Invitae (San Francisco, CA).

### 3.2 Functional assays of mitochondrial enzymes

Assays of PDC, both activated‐dephosphorylated (+DCA) and inactivated phosphorylated (+fluoride), 2‐ketoglutarate dehydrogenase complex (KDC), and dihydrolipoamide dehydrogenase (E3) activities in disrupted blood lymphocytes and cultured skin fibroblasts were measured as previously described.^23^, ^24^ Quantitative oxidative phosphorylation in harvested cultured skin fibroblasts permeabilized with digitonin was measured as described previously.^25^ Spectrophotometric electron transport chain (ETC) complex II‐IV assays in cultured skin fibroblasts were measured as specific donor‐acceptor oxidoreductase activities as described previously.^26^, ^27^, ^28^ Assay of branched‐chain 2‐ketoacid dehydrogenase (BCKDH) complex in disrupted cultured fibroblast by quantitating released ^14^CO_2_ from ^14^C‐leucine decarboxylation was performed at Emory Genetics Laboratory (Decatur, GA).

### 3.3 Expression analysis by Western blotting

Fibroblasts were grown in Advanced DMEM medium (12491‐015 Gibco) with 16% fetal bovine serum (F2442 Sigma) supplemented with 82 U/mL penicillin and 82 μg/mL streptomycin (15140‐122 Gibco), 20 μg/mL uridine (194 763 MP Bio), and 8 ng/mL fibroblast growth factor (F3133 Sigma) in a 37°C incubator with humidified atmosphere of 10% CO_2_. The cells were grown to confluence in a 75 cm^3^ dish. Harvested cells were lysed via two freeze‐thaw cycles and suspended in PBS with 10 μL of 0.1 M PMSF and 0.2 M Leupeptin. For each sample, 50 μg/mL of lysate protein were resolved by SDS‐PAGE and transferred overnight to a PVDF membrane. The PVDF membrane was blocked in 5% milk for 1.5 hours at room temperature (RT) then incubated with primary antibody against PDP1 (492 636 700 Sigma) at 1:600 dilution and against GADPH (TAB1001 Thermo Fisher) at 1:2000 dilution for 1.5 hours at RT. After washing off the primary antibody, the membrane was incubated with 1:1500 dilution of secondary antibody, goat anti‐rabbit IgG HRP (32460 Thermo Scientific), for 1.5 hours at RT. Signals were detected with HyBlot CL autoradiography film (E3018 Denville Scientific Inc.) after applying the SuperSignal West Pico Plus chemiluminescent substrate (34577 Thermo Scientific) to the blot.

### 3.4 Ethics approval and informed consent

The proband was enrolled in the IRB‐approved Manton Center for Orphan Disease Research Gene Discovery Core at Boston Children's Hospital. Informed consent was also obtained from the parents/guardians for additional investigative studies by inclusion in the University Hospitals Cleveland Medical Center IRB‐approved Disorders of Pyruvate Metabolism: Phenotype Genotype Study as well as the IRB‐approved NIH‐funded North American Mitochondrial Disease Consortium research project titled “Natural History and Advanced Genetic Study of PDC Deficiencies,” for additional functional and/or molecular analyses. All procedures followed were in accordance with the ethical standards of the responsible committee on human experimentation (institutional and national) and with the Helsinki Declaration of 1975, as revised in 2000. Informed consent was obtained from the patient for being included in the study.

## 4 RESULTS

### 4.1 Identification of *PDP1* variant by NGS

**Figure 1:**
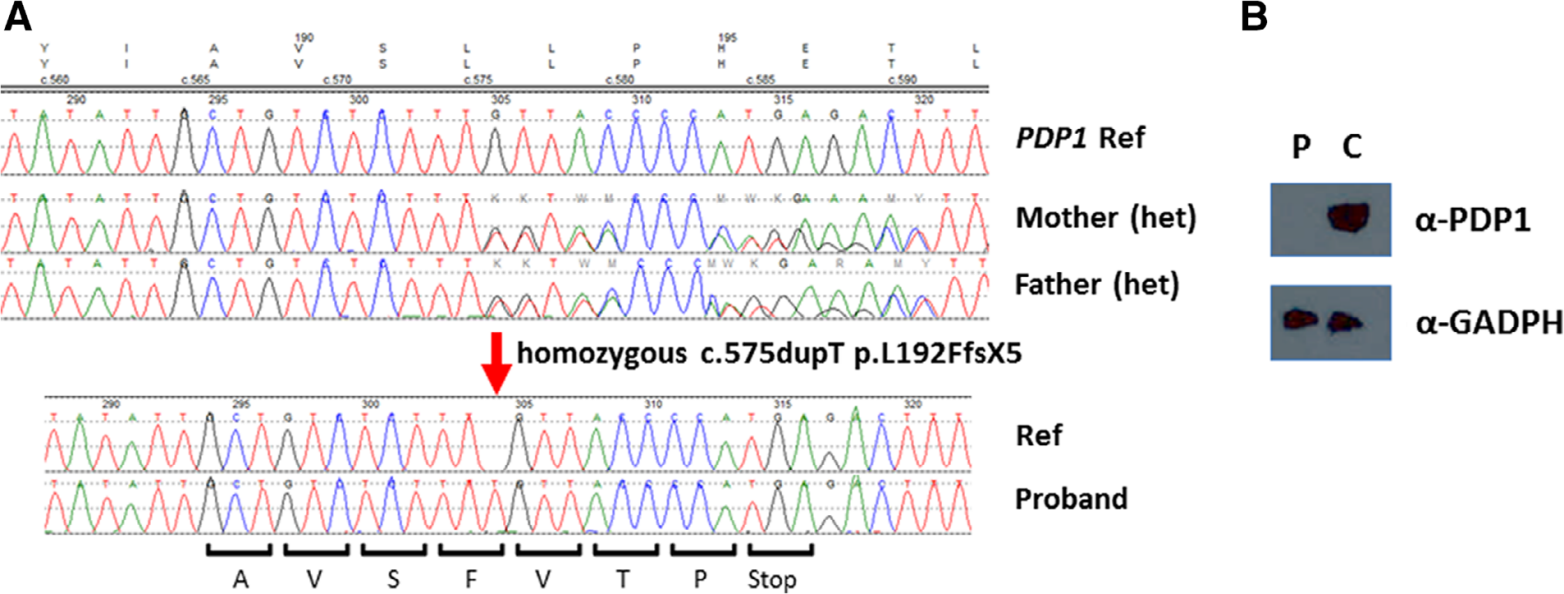
Sequence chromatograms for the genomic DNA region of *PDP1* flanking the mutation (A) and protein expression in patient fibroblasts (B). A, Red arrow shows the duplicated T at c.575 in proband DNA compared with reference sequence. The c.576 position in parents consists of equal amounts of T and G consistent with a carrier status for each parent. B, Immunoblot analysis using antibodies against PDP1 and GAPDH (loading control). C, Patient fibroblast (P) shows absent PDP1 protein expression vs a control sample

The 23‐gene pyruvate metabolism targeted NGS panel identified a novel homozygous c.575dupT p.L192FfsX5 *PDP1* variant in the proband, which was subsequently confirmed by Sanger sequencing and predicted to produce a premature stop codon (Figure 1A). Parents were found to be carriers for the same *PDP1* variant on chromosome (Chr) 8 (Figure 1A), confirming the trans configuration in the proband. Follow‐up protein expression analysis in proband fibroblasts by immunoblotting using antibodies against PDP1 revealed an absent PDP1 protein level (Figure 1B), supporting the pathogenic classification of this variant.

### 4.2 Functional assays

Dichloroacetate (DCA) inhibits the pyruvate dehydrogenase kinases and is often used to identify PDC activation deficiency. Fluoride (F) is used to inhibit pyruvate dehydrogenase phosphatase. Assay of PDC in blood lymphocytes and cultured skin fibroblasts from patient showed decreased activated (+DCA) and inactivated (+F) activity with low PDC/E3 ratio consistent with PDC deficiency (Table 1). In blood lymphocytes, activated and inactivated PDC activities were low at 9% and 25% of control mean, respectively (Table 1). Similarly in cultured fibroblasts, average (n = 2) activated and inactivated PDC activity was 33% and 37% of control mean, respectively (Table 1), with one inactivated value in fibroblasts being low (15%) and another low‐normal (59%). We have observed discordance with successive repeat of fibroblast‐based PDC testing in males in about 5% of cases (n = 70) tested^29^ and the reason for this remains unclear. Activity of dihydrolipoamide dehydrogenase (the E3 component of PDC and another mitochondrial reference enzyme) was normal in both blood lymphocytes and cultured fibroblasts (Table 1). Assay of KDC and BCKDH in cultured fibroblasts from the proband showed normal and low (9%) of control mean activity, respectively (Table 1).

**Table 1:** Summary of functional assays

Enzyme/complex/function	Cell	Activity^a^			
		Case (% mean)	Average (% mean)	Control	
				Mean ± SD, n value	Ref. range
PDC, activated (+DCA)	Lymph	**0.14 (9%)**	**0.80 (33%, n = 2)**	1.63 ± 0.53, n = 596	0.98 to 2.72
PDC, activated (+DCA)	FB	**1.03 (43%)**		2.42 ± 0.88, n = 329	1.26 to 4.42
PDC, activated (+DCA)	FB	**0.57 (24%)**			
PDC, inactivated (+F)	Lymph	**0.13 (25%)**	**0.34 (37%, n = 2)**	0.53 ± 0.23, n = 524	0.22 to 1.09
PDC, inactivated (+F)	FB	0.54 (59%)		0.92 ± 0.63, n = 322	0.19 to 2.30
PDC, inactivated (+F)	FB	**0.14 (15%)**			
E3	Lymph	72.9 (104%)	61.2 (102%, n = 2)	70 ± 16, n = 596	45 to 103
E3	FB	65.7 (110%)		60 ± 29, n = 267	25 to 98
E3	FB	56.7 (95%)			
PDC/E3	Lymph	**0.2 (9%)**	**1.3 (35%, n = 2)**	2.3 ± 0.6, n = 596	1.4 to 3.6
PDC/E3	FB	**1.6 (43%)**		3.7 ± 1.2, n = 198	2.2 to 6.6
PDC/E3	FB	**1.0 (27%)**			
KDC	FB	2.85 (138%)		2.10 ± 1.03, n = 42	0.73 to 4.58
BCKDH	FB		**6.7 ± 0.4 (9%)**	73 ± 22	
OxPhos (pyruvate, malate, and ADP)	FB	42 (108%)		39 ± 6, n = 57	30 to 53
OxPhos (palmitoylcarnitine, malate, and ADP)	FB	45 (155%)		29 ± 4, n = 49	22 to 39
ETC, complex II,III	FB	22.3 (87%)		25.6 ± 4.5, n = 125	17.1 to 33.6
ETC, complex III	FB	49.7 (62%)		79.8 ± 17.7, n = 125	49.7 to 116.7
ETC, complex IV	FB	1.6 (77%)		2.0 ± 0.3, n = 125	1.5 to 2.6
Citrate synthase	FB	50.8 (97%)		52.2 ± 8.8, n = 125	36.3 to 69.9

a PDC, KDC, E3, ETC, CS, and PDC subunit activities were in nmol/min/mg protein, BCKDH activity was in pmol/h/mg protein, and OxPhox activities were in pmol/s/million cells. Final concentrations of Mg^+2^ and Ca^+2^ in the reaction mixes for the PDC (inactivated or activated) assays in fibroblasts and lymphocytes were 2.2 and 0.1 mM, respectively. Low values are shown in bold.

Abbreviations: BCKDH, branched‐chain α‐ketoacid dehydrogenase; CS, citrate synthase, DCA, dichloroacetate; ETC, electron transport chain; F, fluoride; FB, cultured fibroblasts; KDC, 2‐ketoglutarate dehydrogenase complex; Lymph, blood lymphocytes; OxPhos, oxidative phosphorylation—O_2_ consumption assayed in digitonin‐permeabilized fibroblasts (ie, intact cellular mitochondria); PDC, pyruvate dehydrogenase complex; RR, reference range.

Analysis of integrated oxidative phosphorylation in digitonin‐permeabilized proband fibroblasts (ie, with intact cellular mitochondria) showed normal oxygen consumption in the presence of pyruvate, malate and adenosine diphosphate (ADP) as substrates as well as long‐chain acylcarnitine as substrate (Table 1). Changes in oxygen consumption in the presence of pyruvate, malate and ADP reflect composite activity of the mitochondrial pyruvate transporter, production of acetyl‐CoA by PDC, coupled production of NADH, and oxidation of the NADH by Complex I. There were no functional abnormalities of ETC complexes II, III, or IV in homogenized fibroblasts (Table 1).

## 5 DISCUSSION

The clinical presentation of PDC deficiency is highly variable and ranges from fatal congenital lactic acidosis and congenital brain abnormalities including corpus callosum abnormalities (15%‐55%), ventriculomegaly (35%‐85%), and Leigh syndrome (12%‐25%), to relatively mild ataxia or neuropathy with normal cognitive function and long survival.^30^, ^31^, ^32^, ^33^, ^34^ Epilepsy (16%‐57%), hypotonia (46%‐89%), and developmental delay (57%‐83%) are other common findings in subjects with PDC deficiency.^30^, ^31^, ^32^, ^34^ PDC deficiency is sub‐classified into at least three groups, primary‐specific, primary‐generalized, and secondary PDC deficiencies.^29^ Primary‐specific PDC deficiency due to mutations in primary specific genes including *PDHA1*, *PDHB*, *DLAT*, *PDHX*, *PDP1*, *PDP2*, and *PDPR*, constitute 70% to 90% of PDC activity deficiencies, with those due to X‐linked *PDHA1* mutations representing >80% of genetically defined primary‐specific deficiencies.^5^, ^29^ The clinical consequences of defects of primary‐specific PDC gene products are not qualitatively distinct because each of these defects have the common biochemical effect of impairing function of the overall complex.

### 5.1 Therapeutic interventions in PDP deficiency

Life‐long ketogenic diet (KD) use (and avoidance of high carbohydrate diets) is currently the main therapeutic intervention for primary‐specific PDC deficiency with positive outcomes noted in the areas of epilepsy, ataxia, sleep disturbance, speech/language development, social functioning, and frequency of hospitalizations,^30^, ^35^, ^36^, ^37^ but KD can be ineffective in patients with severe brain damage in utero or at birth, and/or lethal in subjects from the other subclasses of PDC deficiency.^22^, ^38^, ^39^ All known patients with PDP deficiency due to *PDP1* mutations have been placed on a ketogenic diet. The patient reported here and others with PDP deficiency due to *PDP1* mutations have shown good response to KD with qualitative improvement of psychomotor development and increased survival.^7^

DCA use has been proposed as an alternate therapeutic intervention for patients with PDP deficiency,^7^ and this is a reasonable option because DCA restores PDC activity in vitro in patients with defective *PDP1* (Table 2). However, given the low DCA‐activated PDC activity in lymphocytes and fibroblasts of the patient reported here (Table 1), the decision was made to use KD as the preferable therapeutic intervention for this patient and follow clinical response.

**Table 2:** Reports of patients with *PDP1* mutations where PDC activity was evaluated in fibroblasts

Genotype	Protein	Elevated blood lactate	L:P ratio	Elevated plasma alanine	PDP1 on western blot	PDC activity in FB		Age of death	Clinical	Ref
						Native (% control mean)	DCA activated (% control mean)			
Hom c.277G > T	E93X	Yes	13 ± 2	Yes	Absent	Low (54%)	Normal (124%)	6 mo	Brain MRI (2 mo of age): normal myelination with no structural lesions identified. MR spectroscopy: increased lactate doublet in the basal ganglia. EM of skeletal muscle: normal mitochondrial size, number and structure. ETC: decreased complex I + III activity relative to CS. On KD	Cameron et al^20^
Hom c.851_853delTTC	L284del	Yes	?	?	Low	Low (30%)	Normal (77%)	?	Hypotonia, feeding difficulties. On KD	Maj et al^9^ ^a^
Hom .851_853delTTC	L284del	Yes	?	?	Low	Low (32%)	Normal (120%)	?	Hypotonia, feeding difficulties. On KD	Maj et al^9^ ^a^
Hom c.575dupT	L192FfsX5	Yes	12	Yes	Absent	ND^b^	Low (33%)	Alive	Developmental delay needing IEP services and epilepsy. On KD	This report

Abbreviations: CS, citrate synthase; DCA, dichloroacetate; EM, electron microscopy; ETC, electron transport chain; FB, fibroblast; Hom, homozygous; KD, ketogenic diet; L, lactate; mo, month; P, pyruvate; PDC, pyruvate dehydrogenase complex; ref, reference.

a Patients are siblings.

b ND, not done. The O_2_ consumption assayed in digitonin‐permeabilized fibroblasts (ie, intact cellular mitochondria) using pyruvate, malate, and ADP as substrates reflecting composite activity of the mitochondrial pyruvate transporter, production of acetyl‐CoA by PDC, coupled production of NADH, and oxidation of the NADH by Complex I, was normal.

### 5.2 Regulation of PDC in PDP deficiency

In contrast to the previously reported cases with biallelic pathogenic *PDP1* mutations where DCA‐activated PDC activity was normal in fibroblasts^9^, ^20^ and lymphocytes,^9^ our case shows low DCA‐activated PDC activity in both lymphocytes and fibroblasts (Table 1) and the reason for this is unclear. The composite activity of the mitochondrial pyruvate transporter, production of acetyl‐CoA by PDC, coupled production of NADH, and oxidation of the NADH by Complex I in intact cellular mitochondria was normal (Table 1). Another metric for activation deficiency due to PDP deficiency is to observe limited or no change between inactivated (+F; “minimum”) and activated (+DCA; “maximum”) PDC activities, with a ratio of 1 representing no change (experience at CIDEM has been that the minimum PDC activity constitutes about 30% to 40% of maximum activity in normal individuals). This is noted in lymphocytes but not in fibroblasts of the PDP deficient patient here where the PDC inactivated to activated ratio was 0.93 (0.13/0.14 with ratio of control means: 0.53/1.63 = 0.33) and 0.39 (average of 0.54/1.03 and 0.14/0.57; with ratio of control means: 0.92/2.42 = 0.38) in lymphocytes and fibroblasts, respectively. The basis for this difference between lymphocytes and fibroblasts is unclear.

The regulatory enzymes pyruvate dehydrogenase (E1) kinase and phosphatase are believed to be specific for PDC, and to the best of our knowledge, there is not an analogous regulatory function for KDC. This is supported by the observation of normal KDC activity in this proband in fibroblasts with biallelic pathogenic *PDP1* mutations (Table 1) as well as in fibroblasts of other reported cases with defective PDP1 where flux through the TCA cycle was unaffected by the low “native” PDC activity (Table 2).^9^ Interestingly, the proband reported here also had low BCKDH activity in fibroblasts (Table 1) with slight elevation of branched‐chain amino acids in plasma without allo‐isoleucine detected and ketoacids in urine, which we speculate might suggest shared regulatory function of PDC and BCKDH by PDP1 at least in fibroblasts but possibly in other tissues or cell types as well. Sequencing and deletion/duplication analyses of *BCKDHA* (on Chr 19), the most common gene mutated in maple syrup urine disease, as well as *BCKDHB* (on Chr 6), *DBT* (on Chr 1), and *PPM1K* (on Chr 4) were all normal (data not shown). Similar to PDC regulation, BCKDH activity is regulated posttranscriptionally by phosphorylation (inactivation) and dephosphorylation (activation).^40^, ^41^ The branched‐chain 2‐ketoacid dehydrogenase phosphatase (BDP) encoded by *PPM1K* is a manganese (Mn^+2^)‐dependent BCKDH complex phosphatase and not a magnesium‐ or calcium‐dependent enzyme.^42^ Furthermore, in contrast to PDP1c, BDP functions as a monomer and the lipoyl prosthetic group on the E2 subunit of BCKDH is not essential for BDP binding or E2‐stimulated phosphatase activity.^42^ The ~16.3‐Mb LOH region on Chr 10 identified in this proband harbors about 116 genes and of those about 41 appear to have function unrelated to BCKDH activity (Table S1). However, we cannot completely rule out the impact of other functionally uncharacterized genes, miRNA or pseudogenes within the LOH region on BCKDH activity. To the best our knowledge, BCKDH activity was not evaluated in other reported cases with confirmed *PDP1* mutations or in the canine model of PDP deficiency due to *PDP1* mutations. Therefore, the suggested potentially shared regulatory function of PDC and BCKDH activities by PDP1 is speculative and remains to be determined.

Finally, the case with the biallelic pathogenic *PDP1* variant (p.E93X) showed decreased ETC complex I + III activity relative to citrate synthase in skeletal muscle (Table 2; quantitative activity data were not reported),^20^ but ETC and oxidative phosphorylation analyses were normal in fibroblasts of the proband reported here, implying variability of cellular energetics due to tissue/cell type, *PDP1* genotype, and/or other modifying genes, which remains to be investigated.

## AUTHOR CONTRIBUTIONS

J. B., G. G., C. H., and D. K. were involved in the biochemical/functional analyses of this patient. L. H., S. T., A. B., E. Y., and G. B. were involved in clinical management of the patient. D. D. assisted with compilation and analysis of clinical data. S. Z. and H. K. S. performed mutational and expression analyses, respectively. J. B. and S. D. were involved in enrolling this patient in the IRB‐approved studies. J. B. wrote the first draft, reviewed, and revised the manuscript, and approved the final version as submitted. All authors approved the final manuscript as submitted and agree to be accountable for all aspects of the work. All authors confirm the absence of previous similar or simultaneous publications.

## CONFLICT OF INTEREST

The authors declare that they have no conflict of interest.

## Supporting information


**Supplementary Figure 1** Supplementary File

**Supplementary Table 1** Supplementary File
