# Supplementary figures and images for: A novel null mutation in the pyruvate dehydrogenase phosphatase catalytic subunit gene (PDP1) causing pyruvate dehydrogenase complex deficiency
Source: JIMD Rep. 2019 Jun 17;48(1):26–35. doi: 10.1002/jmd2.12054 (PMC6606986; doi:10.1002/jmd2.12054)

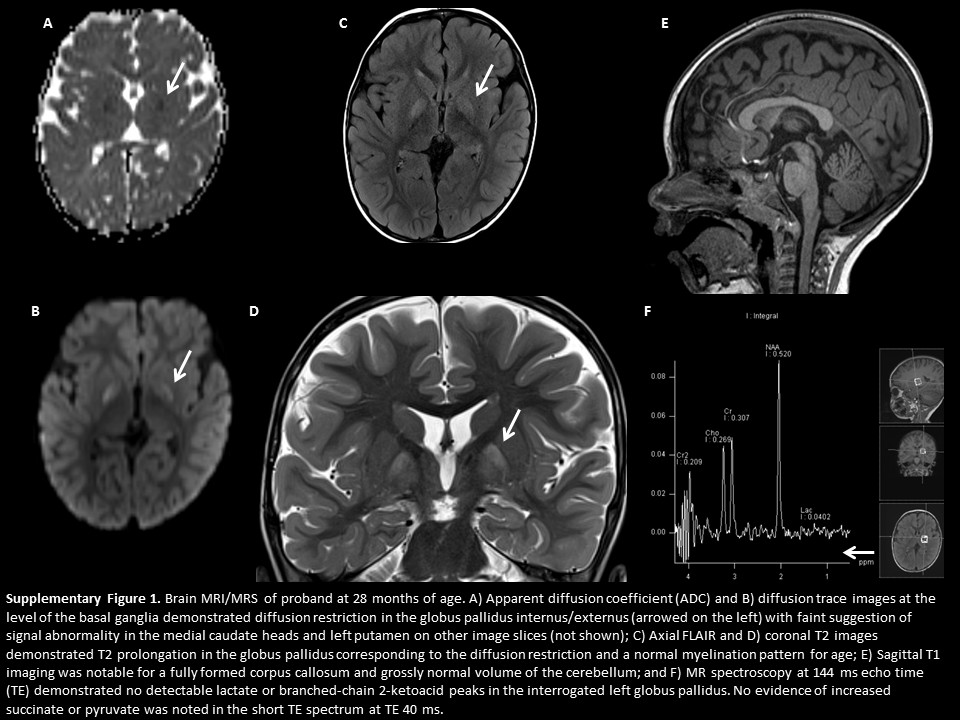

Supplement: Supplementary file 1 — Supplementary Figure 1 Supplementary File [file JMD2-48-26-s001.tif]
